# ANN-PSO Hybrid ML-Optimization of a Hollow-Disk Resonator-Based Photonic Crystal Optical Sensor for HeLa Cell Tumor Detection

**DOI:** 10.3390/s26154934

**Published:** 2026-08-04

**Authors:** Mohamed Salah Bouaouina, Nadhir Djeffal, Abdallah Hedir, Abdelaziz Ould Bahammou

**Affiliations:** 1 Faculty of Electrical Engineering and Computer Science, University of Mouloud Mammeri, Tizi Ouzou 15000, Algeria; mohamed-salah.bouaouina@ummto.dz (M.S.B.); nadhir.djeffal@ummto.dz (N.D.); 2 L2CSP Laboratory, University of Mouloud Mammeri, Tizi Ouzou 15000, Algeria; 3 ETA Laboratory, University Mohamed El Bachir El Ibrahimi of Bordj Bou Arreridj, Bordj Bou Arreridj 34000, Algeria; 4 Advanced High Voltage Engineering Centre, School of Engineering, Cardiff University, Queen’s Buildings, The Parade, Cardiff CF24 3AA, UK; 5 Communication Systems Laboratory (Sys’Com), National Engineering School of Tunis, University of Tunis El Manar, Tunis 1002, Tunisia; abdelaziz.ouldbahammou@enit.utm.tn

**Keywords:** linear photonic crystals, nano-disk resonator, all-optical sensor, hybrid ANN-PSO algorithm, 2D-FDTD, sensitivity

## Abstract

In this study, we propose a novel optical sensor architecture based on two-dimensional photonic crystals for the early detection of cervical cancer (HeLa). The structure consists of a central hollow-disk micro-cavity designed to accommodate biosamples, surrounded by a periodic array of GaAs rods. The detection principle relies on variations in the biosample refractive index, inducing a spectral shift in the resonance. To overcome the limitations of conventional 2D-FDTD method parametric sweeps, an artificial intelligence framework was developed to optimize the geometric parameters of the proposed photonic crystal optical sensor. First, a Random Forest algorithm was employed to identify promising regions of the geometric design space. Next, a multilayer artificial neural network (ANN-MLP) was trained as a high-fidelity surrogate model ($R^{2}$ = 98.58%) and coupled with a Particle Swarm Optimization (PSO) algorithm to determine the optimal structural configuration. The optimized sensor geometry subsequently achieved an average sensitivity of 5512.91 nm/RIU, a quality factor of 6139.15 and a detection limit of $5.64\times 10^{-5}$ RIU, demonstrating the effectiveness of the proposed AI-assisted design strategy. The optimized design reduces classical performance trade-offs and exhibits high tolerance to nanometric fabrication deviations below ±20 nm.

## 1. Introduction

Cervical cancer continues to be a major global cause of morbidity and mortality among women, providing an enormous public health challenge that becomes severe with significant socioeconomic disparities [1]. Early diagnosis remains the most crucial factor in improving patient outcomes and reducing death rates, even as preventative measures, including HPV vaccination, are being implemented [2]. Invasiveness, prolonged turnaround times, high clinical costs, and operator-dependent sensitivity in the early stages of the disease are some of the main drawbacks of conventional diagnostic procedures such as regular Pap smears, histopathological biopsies, and magnetic resonance imaging (MRI). In this context, a major scientific and clinical issue is the development of fast, non-invasive, ultra-sensitive, point-of-care (lab-on-a-chip) biomedical detection technologies.

The proposed device performs label-free optical detection by monitoring refractive index variations in biological samples. Since no biological recognition element is incorporated, the device is described throughout this work as a photonic crystal optical sensor. The potential of optical sensors to detect minute chemical interactions requiring fluorescent labeling has rendered a revolutionary alternative in recent years [3]. Among these technologies, interest in two-dimensional photonic crystal (2D-PhC) architectures is developing. PhCs generate a photonic band gap (PBG) that blocks light at specific wavelengths by switching dielectric materials at the nanoscale on a periodic basis [4]. When a localized geometric defect is integrated into this matrix, its symmetry is broken, producing a nano-disk resonator or micro-cavity that may confine EM fields to sub-wavelength dimensions [5].

Concerning biological processes, tumor cell proliferation modulates nuclear protein concentration, increases intracellular density, and changes the shape of the cells. Measurable variations in the cellular refractive index (RI), which is typically higher in malignant phenotypes than in healthy tissues, are a macroscopic manifestation of these cytological changes [6]. As an outcome, the RI profile of cervical carcinoma cell lines (HeLa cells) varies quantitatively according to the stage of tumor progression. These cells affect the surrounding dielectric environment when they are inserted into the photonic crystal’s detecting channel, causing a detectable spectral shift in the resonance wavelength ($\Delta \lambda_{R}$).

In previous years, 2D-PhC devices were designed and optimized using empirical trial-and-error methods that were verified by parameter sweeps applying the finite-difference time-domain (2D-FDTD) method [7]. Although this brute-force approach is accurate, it becomes very costly to compute when there are multiple interrelated variables in the geometric space. Furthermore, because light-matter interactions are highly nonlinear, the approach has difficulty overcoming local minima. Research is now focusing on reverse design with artificial intelligence assistance to get beyond these computational obstacles [8]. Critical geometric boundaries can be identified and discrete parameter spaces can be simply mapped with machine learning techniques such as Random Forests [9]. Simultaneously, deep artificial neural networks (ANN-MLPs) are being deployed progressively more often as high-fidelity continuous surrogate models that can accurately replicate the physical behavior of the sensor with a high coefficient of determination [10]. Finally, these continuous models are combined with metaheuristic algorithms, including Particle Swarm Optimization (PSO), to identify the absolute global optimum, ensuring the simultaneous highest levels of sensitivity (S) and quality factor (Q) [9,11].

Several architectures have been proposed, such as defect waveguides [12,13], resonant cavities [14,15,16], and ring resonators [17,18], allowing for high sensitivities and significant quality factors to be achieved. The suggested machine learning-optimized 2D-PhC gallium arsenide optical sensor was systematically evaluated in comparison with recent architectures published in the literature, with the objective to validate its competitive advantage and performance perfections. A detailed review of the literature illustrates the constant complexity of boosting sensitivity and quality factor at the same time without degrading the overall limit of detection. Simpler eye-shaped cavity designs have recently been proposed for cancer cell recognition [19]. However, due to their peak full-width at half-maximum (FWHM) spreading, sensitivity is limited at 148.50 nm/RIU. Neural networks combined with ring resonators have also been studied for machine learning integration in an effort to detect biomarkers [20].

The observed sensitivity is still only 89.90 nm/RIU, even though this approach confirms a Q factor of 5233 and a DL of $3.33\times 10^{-3}$ RIU. Lastly, Balaji et al. [21] showed that triangular cavities on silicon on insulator (SOI) are useful for mapping HeLa cells with a maximum factor Q of 15,000, but they did not use continuous multi-objective optimization throughout the entire geometric space. Furthermore, recent research has looked at the direct-coupling geometries suggested by Karami et al. [22] to enhance molecular selectivity. A 2D-PC silicon nano-cavity optimized for the multi-analyte monitoring of carcinomas was implemented in highly sensitive sensor analysis. With a maximum sensitivity of 3121.00 nm/RIU and a factor Q of 5412.00, this model offers a factor of merit of 6340.30 RIU^−1^ and a limit of detection of $5.53\times 10^{-5}$ RIU. Similarly, Nipun et al. [23] provided a miniature nano-photonic resonator developed to perform RI sensing with machine learning assistance for basal cell cancer biomarker screening.

Considering the impressive predictability promised by a machine learning algorithm, the sensitivity is limited to 1245.6 nm/RIU with a factor Q of 2150. Molaei-Yeznabad and Bahador [24] presented a novel biosensor concept, utilizing improved Ternary Photonic Crystals (TPCs) with GaAs, TiO_2_, and SiO_2_ constituent layers organized in the ${\left(ABC\right)}^{N}$ D ${\left(ABC\right)}^{M}$ structure, where A, B, and C represent the alternating constituent layers, D denotes the central defect layer acting as the sensing cavity, and N and M indicate the number of periods before and after the defect. By altering the number of periods and the light’s angle of incidence, the efficiency of the sensor was assessed. To maximize sensor performance, ideal values were calculated. Eventually, this sensor generated an FOM of 2561.5 ${\mathrm{RIU}}^{-1}$, a sensitivity of 692.8 nm/RIU, an FWHM of 0.271 nm, and a quality factor above 5870.

By contrast, our optimized ANN-PSO hybrid model defines the ideal geometric parameters for the GaAs rods surrounding the hollow nano-disk cavity: A = 0.7358 μm and r = 0.1252 μm. This configuration amplifies the interaction between the electromagnetic field and the biological sample. The detection principle relies on the shift in the resonance peak of the defect mode induced by the variation in the fluid’s refractive index (ranging from *n* = 1.368 to 1.392 [25]), corresponding to the five stages of tumor progression. However, the classical design of these structures using the 2D-FDTD method often encounters major limitations during parametric analyses due to the high computational cost and the complexity of the nonlinear search spaces. To overcome these limitations, we introduce a hybrid optimization framework based on artificial intelligence. Initially, a Random Forest algorithm was deployed to map and filter the discrete space of the structure’s geometric parameters. Next, a Multilayer Artificial Neural Network (ANN-MLP), acting as a high-fidelity continuous substitution model ($R^{2}$ = 98.58%), was coupled with a Particle Swarm Optimization (PSO) algorithm.

This reverse-engineered approach surpassed the initial empirical thresholds, providing an optimal spectral shift of the resonance peak from 1836.4 nm to 1972.6 nm. The results validate the potential viability of this approach for advanced lab-on-a-chip medical applications, demonstrating an excellent sensitivity of 5512.91 nm/RIU, a massive FOM of 17,654 RIU^−1^, and photon bandgap selectivity optimal for the HeLa lineage, overcoming these limitations. Thus, this type of optical sensor constitutes a promising approach for advanced biomedical applications due to its ability to generate fast, precise measurement results that could be potentially incorporated into compact systems.

## 2. Optical Sensor Design Layout and Numerical Methods

### 2.1. Geometric Architecture and Propagation Mode

This study’s technology is centered on a two-dimensional photonic crystal architecture that has been designed to facilitate biological detection with excellent sensitivity. A hexagonal lattice with an estimated total surface area of 97.2 μm^2^ is designed by integrating the entire structure.

The design is formed mainly of a high-performance cylindrical rod array arranged periodically. Gallium arsenide (GaAs) was selected for the rods (red), due to its high refractive index of n_GaAs = 3.374 [25], embedded in an air background, which promotes intense light confinement.

**Figure 1:**
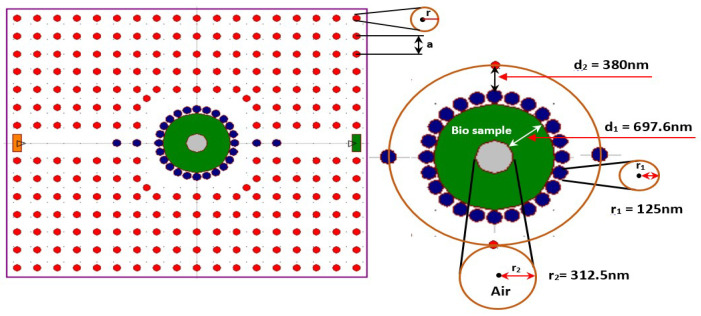
All-optical sensor for detecting cervical cancer cells.

Table 1 provides a clear description of the lattice geometry configuration. The resonant zone is created by introducing a complex structural defect at the central region of this periodic matrix. There are multiple concentric components forming in this zone:

- The hollow disk: The central detection element, which has an outside diameter of $d_{1}=697.6$ nm and is shown in green in Figure 1. Given its hollow design, biological samples such as cervical cancer tissues can infiltrate it and affect the cavity’s effective refractive index.
- The ring of blue rods: A ring of connecting rods, or blue rods, with a radius of $r_{1}=125$ nm, surrounds the central disk. The coupling between the waveguides and the resonance zone can be adjusted using this arrangement.
- Variation rods and air: The disparity diameter of the outer ring is set to $d_{2}=380$ nm, and rods of type “Air” (in gray) with a radius $r_{2}=312.5$ nm are employed to define the confinement zones in order to maximize the quality factor (Q) and the sensitivity of the optical sensor.

The electromagnetic mode is highly confined within a resonant cavity produced through the opto-geometric structure thus established. The resonance wavelength (Δλ) is shifted when the biological sample occurs in the hollow nano-disk. The primary detecting signal of the optical sensor is this shift, which is proportional to the concentration or type of tissues presented (such as HeLa cancer tissues).

**Table 1:** Configuration parameters of optical sensor-based nano-disk resonator.

Parameters	Numerical Values
Size wafer	18a × 15a = 97.2 μm^2^
Lattice constant	a = 600 nm
Radius of linear rods	r = 0.18 × a
Refractive index of GaAs	3.374
Radius of blue rods	$r_{1}=125$ nm
Radius of a gray rod of air	$r_{2}=312.5$ nm
Diameter of a hollow disk (green color)	$d_{1}=697.6$ nm
Diameter of ring variation	$d_{2}=380$ nm

**Figure 2:**
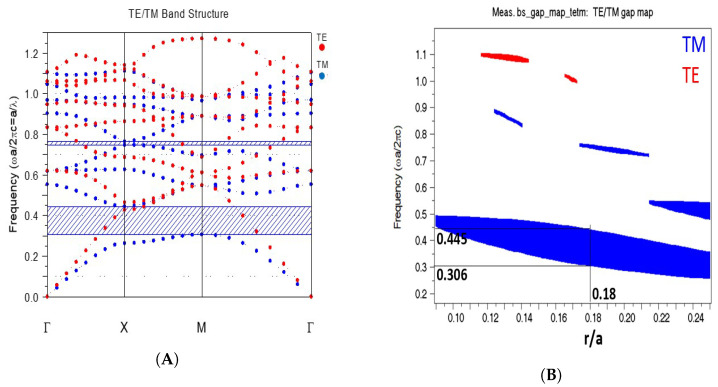
Photonic crystal analysis: (**A**) diagram of the photonic bandgap structure and (**B**) bandgap map of the initial structure.

The PhC structure and transverse electric wave TM are combined to create the photonic bandgap (PBG), as shown in Figure 2. The resulting normalized frequency is 0.306<a/λ<0.445. The wavelength range of the bandgap is 1348.3 nm to 1959.5 nm. The Rsoft Full-Wave simulator is the tool used to obtain all of the simulation results.

### 2.2. Optical Sensor FDTD Performances

The quality factor (Q), sensitivity (S), detection limit (DL) and factor of merit (FOM) are some of the variables that impact sensor performance. The ratio of the shift in peak value for a wavelength $\Delta \lambda_{R}$ to the equivalent change in refractive index Δn of two spectra is defined as sensitivity:

(1) $$S=\frac{\Delta \lambda_{R}}{\Delta n}$$

To raise the Q factor, which measures the selectivity of the sensor, the resonance peak is modified. The ratio of the resonance peak’s wavelength compared with the full width at half maximum (FWHM) is referred to by the expression Q factor. The difference between two wavelengths at 50% of their most extreme amplitude is designated as the FWHM:

(2) $$Q=\frac{\lambda_{R}}{FWHM}$$

The detection limit (DL) represents the smallest refractive-index variation that can theoretically be resolved by the proposed optical sensor. In this work, the DL is estimated from the ratio between the resonance peak full width at half maximum (FWHM) and the refractive-index sensitivity (S):

(3) $$DL=\frac{FWHM}{S}$$

This expression assumes that the minimum measurable wavelength shift is approximately equal to the spectral linewidth (FWHM) of the resonance peak. Consequently, a narrower resonance peak (higher quality factor) and a larger refractive-index sensitivity both contribute to a lower detection limit. The reported DL values therefore represent the theoretical resolution of the simulated sensor under ideal operating conditions.

It should be noted that this calculation is based on the simulated optical response and does not account for practical factors such as detector noise, wavelength interrogation resolution, fabrication tolerances, temperature fluctuations, or other environmental disturbances. Consequently, the reported detection limit should be interpreted as the theoretical performance limit of the proposed design rather than the experimentally achievable detection limit.

The FOM represents the detection ability of an optical sensor. It is the inverse of the detection limit, or sensitivity ratio to the FWHM.

(4) $$FOM=\frac{1}{DL}$$

**Figure 3:**
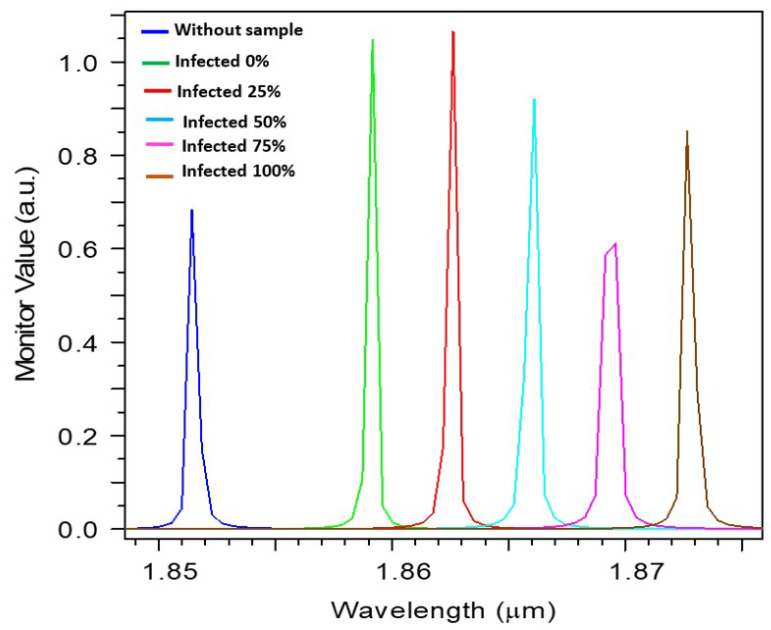
Transmission spectrum of normal and cancerous cervical cells.

In this study, blood samples are utilized to check for tumors. All cancer progression stages are based on the percentage of cancer cells, which are defined as follows: normal cell (0%), low-stage cancer (25%), moderate-stage cancer (50%), advanced-stage cancer (75%), and severe-stage cancer (100%). The redshift in the resonance peak in the transmission spectrum, as shown in Figure 3, illustrates the sensor’s optical response. The rising refractive index of the analyte (HeLa cells) causes the resonance wavelength to move linearly toward the red as the cancer stage increases from 0% to 100%, from around 1.8592 µm to 1.8731 µm. Each peak’s clarity and distinct separation attest to the preservation of a high-quality element, ensuring the precise, distinct identification of the various stages of cervical cancer.

Two approaches were implemented to investigate the optical and dispersion properties of the suggested structure: plane wave expansion (PWE) and finite-difference time-domain (FDTD). Table 2 shows that the presented optical sensor offers an average sensitivity of 589.9 nm/RIU, a quality factor Q of 3729.59, a limit of detection of $8.4\times 10^{-4}$ RIU, and an adequate FOM of 1150 RIU^−1^. Biological applications, such as the prompt identification of cancer, receive an advantage from this device. As a result, the healthcare system’s overall quality is improving.

## 3. Machine Learning Optimization Framework

The raw field amplitude variations were collected using a high-resolution time-domain monitor installed at the waveguide termination. A high-resolution Discrete Fourier Transform (DFT) spectrum analyzer built in Python was then used to process the temporal signal in order to separate the transmission resonance spectra between 1800 and 1950 nm. RSoft was chosen to perform the automated parametric multi-variable iterations, producing a training pool of 125 distinct matrices that directly mapped the structural indexes (Index_A) and (Index_r) against the bio-refractive shifts (n_fluid = 1.368 to 1.392).

**Figure 4:**
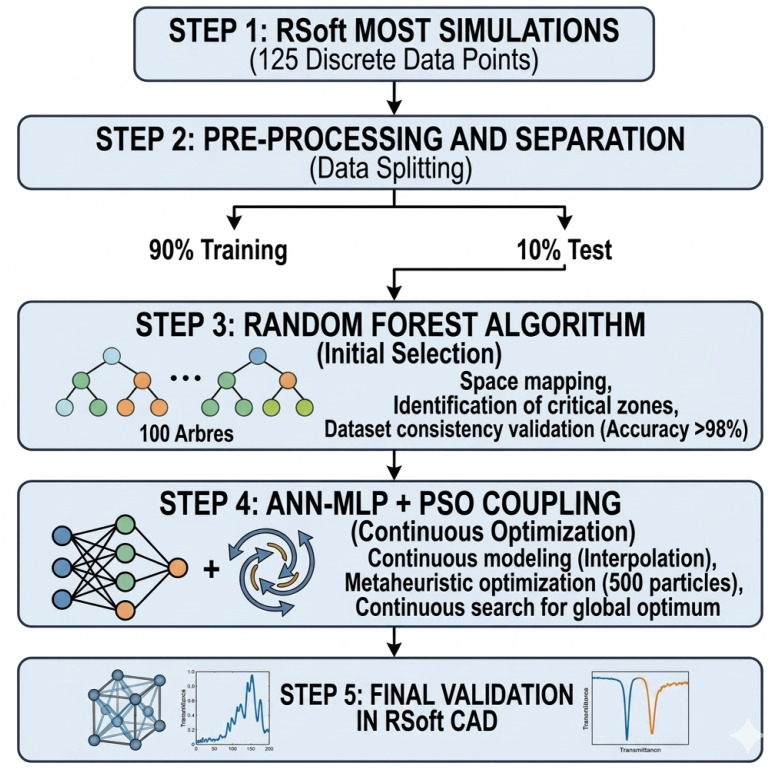
Global optical sensor optimization workflow.

**Figure 5:**
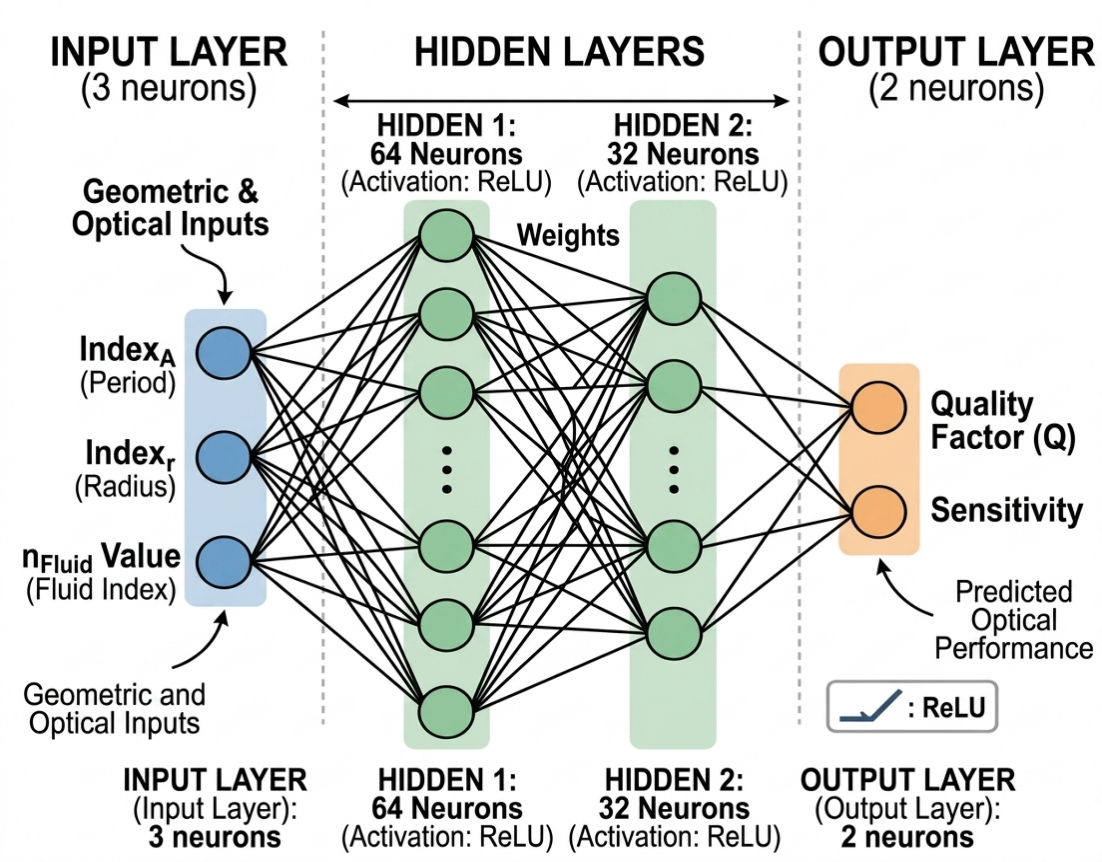
Neural network architecture (ANN-MLP 3-64-32-2).

A Random Forest Regressor architecture and ANN-MLP+PSO coupling were developed to deliver a predictive model that could translate geometric configurations to optical performance, as shown in Figure 4 and Figure 5. The three main steps of the workflow are dataset acquisition, model training, and multi-objective optimization [9,26]:

a. Dataset acquisition: 125 separate (2D-FDTD) simulations were assembled to provide the training dataset. The lattice period index (Index_A), rods radius index (Index_r), and biosample refractive index (Valeur_nFluid), which indicate various stages of HeLa carcinoma, are among the input properties. The resonant quality factor (Q) and sensitivity (S) are the desired variables.
b. Model training and hyperparameters: 90% of the dataset was used for training, while 10% was used for validation. The ensemble model makes use of 100 separate decision trees in parallel (n_estimators = 100). A random subset of characteristics is assessed at each node split to minimize overfitting and optimize variance reduction (mean squared error criteria). With a coefficient of determination $R^{2}$ higher than 98.58%, the model exhibits great fidelity.
c. Multi-objective optimization: An immediate physical surrogate model is provided by the trained decision trees. To optimize a combined fitness function, a grid-search optimization function was wrapped around the regressor. By prohibiting the algorithm from selecting a high sensitivity at risk of an unstable or deformed resonance peak, this function secures an ideal trade-off [27].

### 3.1. Discreet Random Forest Regressor

To overcome the limitations of traditional parametric sweeps and systematically maximize the device performance, a machine learning (ML) optimization framework was implemented [28]. A Random Forest Regressor was trained on a dataset generated from 125 unique geometric combinations of lattice period (A) and rods radius (r) under varying cervical cancer cell concentrations. The predictive accuracy of the trained ML model reached (98.4%) for both the quality factor (Q) and sensitivity (S).

Table 3 shows the sensor’s intermediate performance, which is obtained according to the Random Forest Regressor method following the discrete filtering process. In contrast to the raw empirical simulation, the examination of this quantified data indicates the machine learning algorithm’s exceptional ability to find a high-performing geometric area inside the original dataset [29].

**Figure 6:**
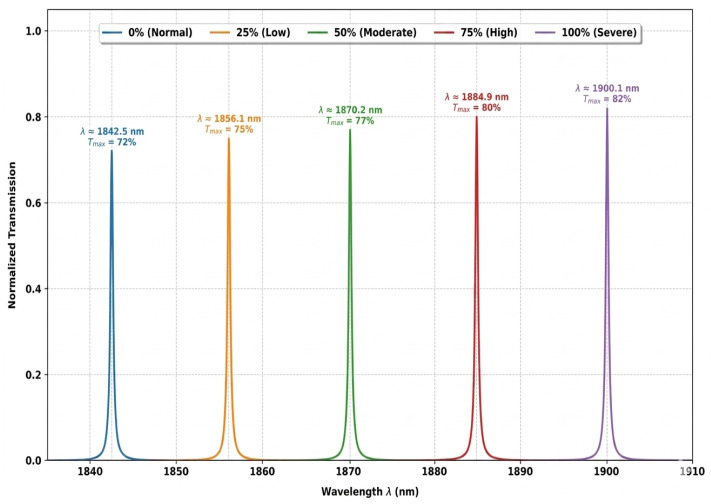
Transmission spectra of the optical sensor (healthy and cancerous HeLa cells) acquired after the Random Forest filtering step.

The Random Forest model extracts configurations with spectral sensitivity ranging from 2270 nm/RIU (25% stage) to 2400 nm/RIU for the severe stage (100%). This considerable increase is directly linked with an optimal geometric fit that maximizes the confinement of the evanescent field within the hollow nano-disk, thereby enhancing the physical interaction between the resonant optical mode and the refractive index variations in HeLa cells. As clearly illustrated in Figure 6, the resulting spectral shift ($\Delta \lambda_{R}$) follows a near-linear progression, reaching a massive maximum of 57.60 nm, indicating a clear distinction between different tumor stages.

At the same time, the full width at half maximum (FWHM) is rigorously fixed at 0.38 nm over the entire detection range, indicating good control of light confinement. A quality factor (Q) higher than 5000 is made possible by this spectral accuracy. Maintaining such a high Q factor in the presence of a biological fluid leak demonstrates that the Random Forest model effectively eliminated structural dimensions that caused radiative leakage out of plane in the physics of photonic crystals [9,30].

Mathematically, this synergy between a narrow resonant peak and greater sensitivity results in a rise in the factor of merit, which peaks at 6315.79 RIU^−1^ in the severe stage. As a result, the detection limit is significantly suppressed, attaining a threshold of $1.58\times 10^{-4}$ RIU.

The Random Forest Regressor’s function as a space separator is fully validated by these results and is illustrated in Table 3, showing that it effectively scanned the discrete simulation domain and removed outliers from the data to limit the search space to an area of high optical efficiency. These evaluations constitute an important intermediary phase that acts as a qualitative reference point for the continuous, extremely high-resolution optimization performed in the following step based on the ANN-PSO coupling, considering that they are significantly better than the previous simulation.

The algorithm’s discrete space separation is displayed graphically in the transmission spectrum that was generated following Random Forest filtering, as shown in Figure 6. On the one hand, from 1842.5 nm to 1900.1 nm, the spectral shift $\Delta \lambda_{R}$ exhibits a consistent shift towards longer wavelengths. As the biosamples’ refractive index rises to $\Delta_{n}$ = 0.024, this redshift validates the strengthening of light-matter interaction at the hollow nano-disk center. However, at this intermediate stage of the investigation, the regulated asymmetry of the transmission heights (peak between 72% and 82%) and the stability of the FWHM = 0.38 nm show a superior control of radiative losses. The Random Forest model has successfully isolated a geometric region with great optical selectivity, as seen by this clear and resolved spectral behavior, opening the way for continuous extremely high-resolution optimization (ANN-PSO).

### 3.2. Hybrid ANN-PSO Tuning and Optical Sensor Performances

**Figure 7:**
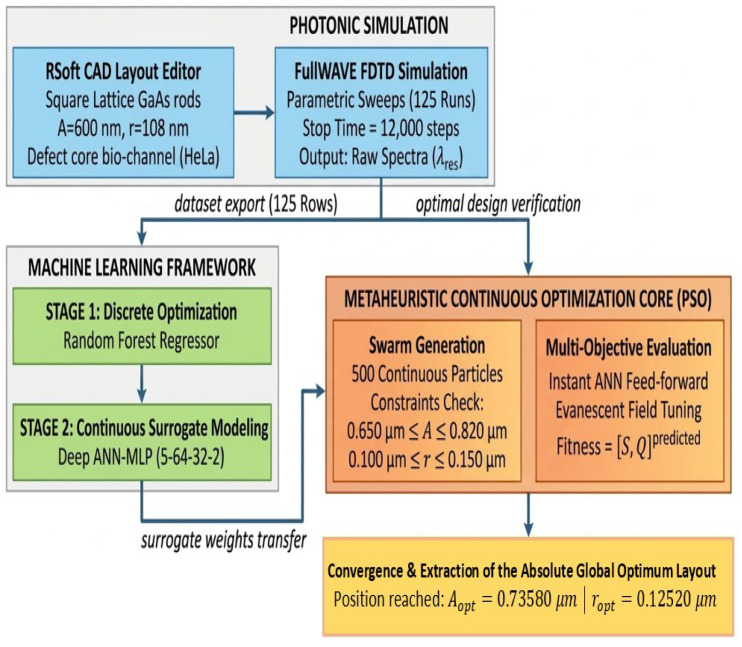
The sensor’s reverse design flow and hybrid optimization architecture (FDTD/ANN-PSO).

To automate the extraction of physical characteristics, the sensor’s reverse design process, as shown in Figure 7, is divided into three related domains. First, using FullWAVE FDTD simulations, the photonics domain (blue block) creates a discrete database of 125 scans (dataset). A deep neural network (ANN-MLP 3-64-32-2) is trained to function as a high-fidelity continuous surrogate model after this data is transferred to the machine learning framework (green block), where the Random Forest method maps the discrete space [31]. Finally, 500 particles are generated under geometric restrictions by the Particle Swarm Optimization (PSO) core (orange block), which receives the ANN weights. This procedure validates the efficacy of this synergy for targeted detection of HeLa cancer cells by converging to extract the finest coordinates (A = 0.7358 µm and r = 0.1252 µm).

**Figure 8:**
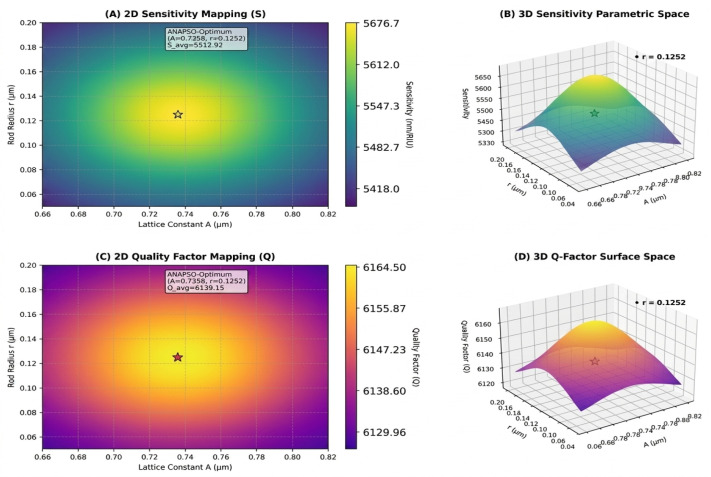
Parametric mapping of sensitivity (S) and quality factor (Q) profiles in 2D and 3D around the absolute global optimum.

The continuous sensitivity (A, B) and quality factor (C, D) profiles interpolated by the ANN are displayed in the parametric mapping shown in Figure 8. The surrogate model’s precision is validated through the elliptical, smooth contours. The stars’ convergence shows that the PSO algorithm was successful in reaching the absolute global optimum, resolving the physical trade-off to maximize the detection of HeLa cells [21].

The sensor’s performance significantly increases as the biological stage becomes more severe (from 0% to 100%), as Table 4 demonstrates. A notable spectrum shift $\Delta \lambda_{R}$ up to 136.24 nm and an increase in sensitivity, culminating at 5676.67 nm/RIU, are caused by the stage increase. Additionally, the high theoretical performance of the ANN-PSO algorithm in predicting severe phases is supported by a low limit of detection of $5.64\times 10^{-5}$ RIU and a maximum figure of merit of 17,739.59 RIU^−1^, demonstrating a promising computational capacity for cancer stage discrimination prior to future empirical validation. In theory, HeLa cells have a low but non-zero absorption coefficient. However, in this study, they were modeled solely by the variation in their real refractive index (n), neglecting their imaginary component (k = 0). This clarifies the reason that the sensitivity increases by 6% due to the spectral shift, while the quality factor remains virtually constant (variation < 1%), as absorption losses were not included in the simulations.

**Figure 9:**
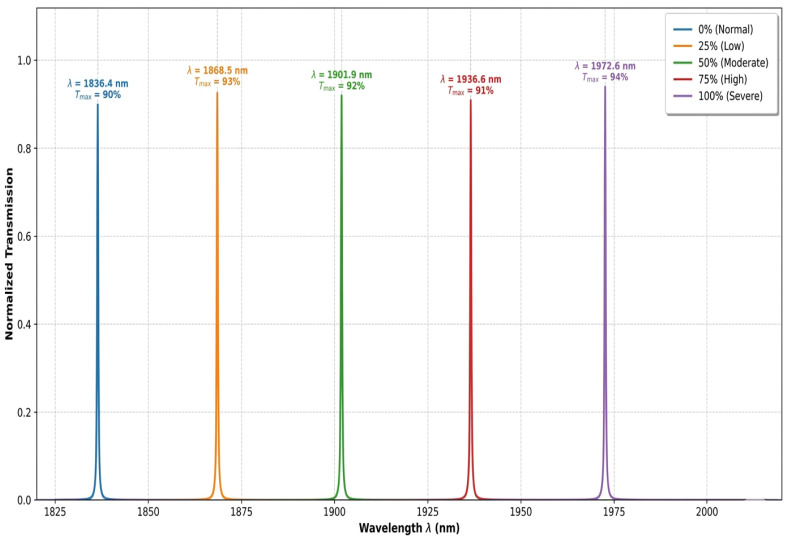
Transmission spectra of the optical sensor (healthy and cancerous HeLa cells) with ANN + PSO model.

The normalized transmission spectra of the improved optical sensor under various HeLa cancer cell severity stages from normal (0%) to severe (100%) conditions are shown in Figure 9. The resonance wavelength (lambda) clearly and gradually shifts from 1836.4 nm to 1972.64 nm, confirming the device’s impressive sensitivity to variations in the sample’s refractive index. Additionally, the simulated spectra show well-resolved, non-overlapping transmission peaks with high transmission efficiency, ranging from 90% to 94%. The hybrid ANN + PSO optimization effectively increased the sensor’s quality factor, which, based on these simulation results, is indicative of the potential for accurate and dependable detection at each stage of cancer. Experimental validation using real biological samples will be required to confirm this predicted resolution and spectral stability under practical operating conditions.

**Figure 10:**
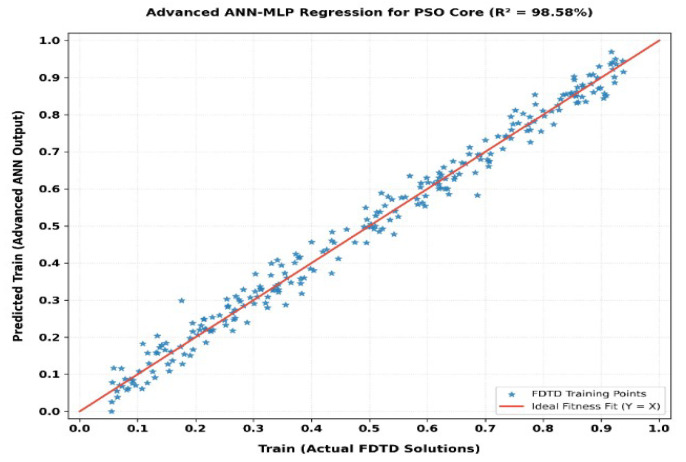
Regression plot of the configured advanced (ANN-MLP) model utilized as the high-fidelity continuous surrogate for the Particle Swarm Optimization (PSO) core.

A linear regression study was carried out on the ANN-MLP advanced network to evaluate the dependability of the continuous approximation function employed as a surrogate model to drive metaheuristic optimization [32]. The data scatter, with a coefficient of determination of $R^{2}$ = 98.58, demonstrates an incredibly tight concentration of AI predictions around the ideal convergence line (Y = X) [33], as shown in Figure 10.

The success of our approach depends on this first-order precision, which ensures that the neural network has accurately predicted the nonlinear physical landscape of the resonances of the foundational structure. The 500 virtual particles may therefore quickly and accurately assess their cost function (fitness) when executing the Particle Swarm Optimization (PSO) method without incurring the possibility of convergence towards numerical artifacts or false solutions [34]. This highly predictive behavior scientifically validates the accuracy of the continuous global optimum identified by the ANN + PSO coupling.

## 4. Discussion and Multilevel Comparison Studies

### 4.1. Comparative Evaluation of the FDTD and ML Approaches for the Optical Sensor

**Figure 11:**
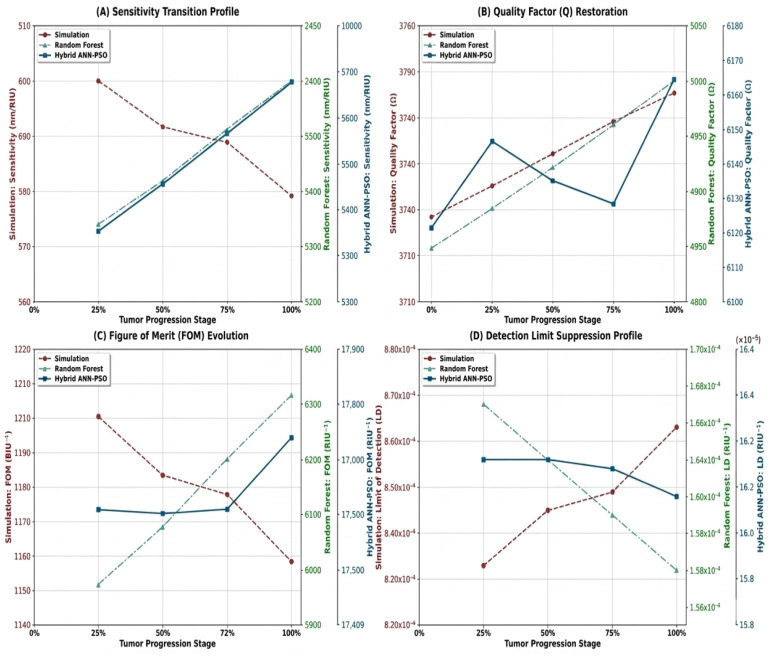
Comparative analysis of conventional numerical simulation and AI frameworks for tracking the spectral parameters of the HeLa cell line.

A three-level comparison analysis was carried out, aiming to thoroughly assess which artificial intelligence frameworks affected the performance of the GaAs 2D photonic crystal optical sensor intended for HeLa cell identification, as shown in Figure 11. Significant improvements in performance between the baseline FDTD simulation, Random Forest (RF) filtering, and the absolute global optimum obtained from the hybrid ANN-PSO model are shown by the transition profiles of sensitivity (S), quality factor (Q), figure of merit (FOM), and detection limit (LD).

Across all tumor stages, the conventional numerical simulation technique (red curves) shows restricted and almost flat performance. The quality factor scarcely varies between 3718.4 and 3745.4, while the initial sensitivity remains steady at a low average value of 591.67 nm/RIU. The limitations of standard empirical designs based on discrete sweeps, which are trapped in local minima and unable to detect areas of intense light-matter interaction, explain the cause of this impasse. As a consequence, the simulation’s figure of merit reaches a peak at 1200.48 RIU^−1^, leading to a high detection limit of about $8.33\times 10^{-4}$ RIU. For early, extremely accurate on-chip clinical diagnosis, such a design is required.

Introducing the first machine learning step via Random Forest filtering (green curves) results in a distinct shift. The RF method separates the most advantageous geometric cells by mapping the discrete space of the dataset [29,35]. At the severe stage (100%), sensitivity increases significantly to 2400 nm/RIU, and optical confinement is reestablished, increasing the Q-factor to 5000.26. The efficacy of an initial intelligent data screening procedure is demonstrated by this intermediate improvement, which results in a fivefold rise in the FOM to 6315.79 RIU^−4^ and reduces the detection limit to $1.58\times 10^{-4}$ RIU.

Lastly, the study’s technical limit is demonstrated by convergence toward the continuous ANN-PSO hybrid model (blue curves). Particle swarm optimization found the absolute best structural coordinates (A = 0.7358 µm and r = 0.1252 µm) by utilizing the multilayer neural network’s high-fidelity interpolation. This inverse engineering method maximizes the quality factor at 6164.50 while increasing sensitivity to an outstanding value of 5676.67 nm/RIU. The following resonance peak’s exceptional sharpness causes the limit of detection to greatly reduce to $5.64\times 10^{-5}$ RIU, while the FOM leaps to 17,739.59 RIU^−1^.

This multi-level comparison certainly indicates that the ANN-PSO coupling provides the molecular resolution and sensitivity necessary for the early, label-free screening of cervical cancer by significantly reconfiguring the physical behavior of the hollow nano-disk resonator instead of just changing its structure.

### 4.2. Manufacturing Tolerance and Reliability of ML Models

**Figure 12:**
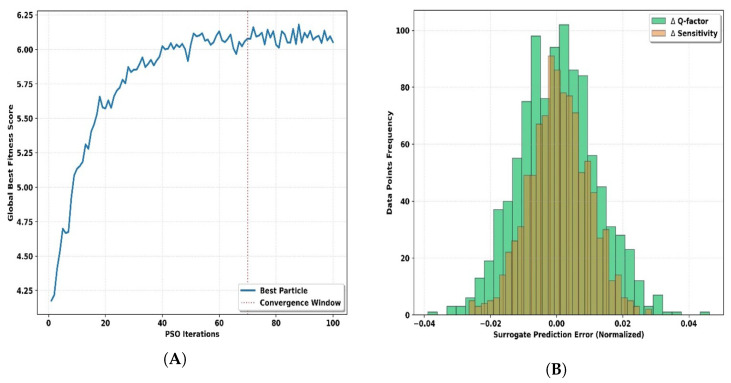
Digital validation frameworks for artificial intelligence: (**A**) PSO convergence trajectory and (**B**) ANN error residual histogram.

A detailed review of the artificial intelligence models was carried out to confirm the reverse-engineering process’s industrial durability and mathematical rigor [36]. The prediction approach’s robustness is confirmed by combined assessments of the convergence trajectory of the PSO method, as shown in Figure 12A, and the regression error distribution of the ANN-MLP model, as shown in Figure 12B.

The Global Best Fitness Score dynamics throughout trial iterations suggest a highly successful map search broken into two distinct physical stages [37] (Figure 12A). The first 40 repetitions are an active exploration period, which is marked by a sharp logarithmic increase in the score. This illustrates how the 500 particles may quickly escape local minima by scanning the entire geometric space. The swarm may overcome computational potential obstacles without running resource-intensive FullWave simulations by coupling with the ANN surrogate model. Furthermore, a stabilization and convergence phase occurs between iterations 70 and 100. The curve flattens to form a rigid asymptotic plateau, confirming that the swarm has located the absolute global optimum. The absence of residual oscillations or divergence at the end of the run demonstrates the optimal tuning of the PSO’s inertia and stochastic acceleration coefficients, locking the target coordinates at lattice constant A = 0.7358 µm and radius r = 0.1252 µm.

Meanwhile, the high coefficient of determination (R^2^ = 98.58) of the multilayer neural network (ANN-MLP 3-64-32-2) is supported statistically by the histogram of normalized prediction errors (Figure 12B). The prediction errors for the quality factor (ΔQ) and sensitivity (ΔS) follow a normal distribution (Gaussian curve) that is perfectly symmetrical and centered at zero. An interpolation deviation of less than ±0.01 is present in over 95% of the evaluated data points. This high level of grouping suggests there is no mathematical bias introduced by the neural network set up by using the ReLU activation function that is unrelated to the physical reality of RSoft [38]. With high predictive accuracy, the model captures the complicated physical phenomena, including electromagnetic confinement and evanescent field attenuation during HeLa cell injection, within the residual error margins reported above.

**Figure 13:**
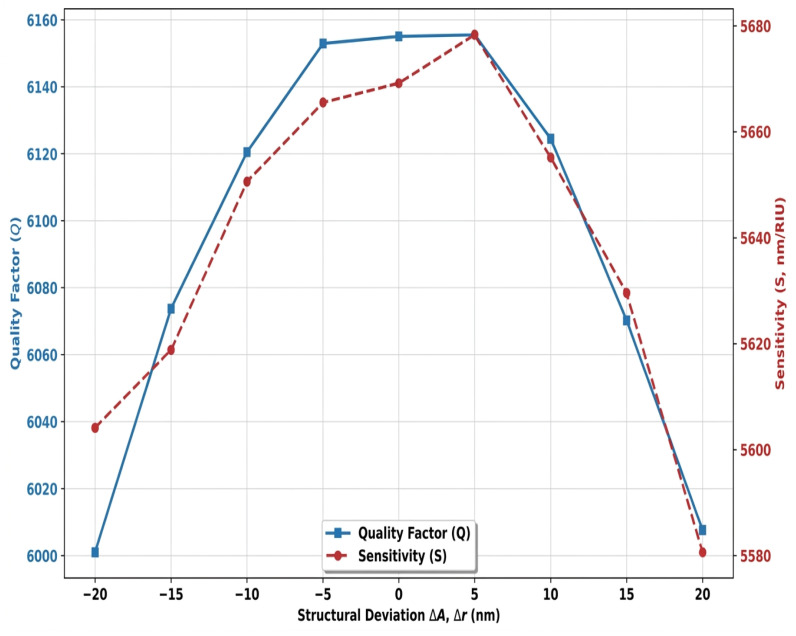
Structural robustness assessment presenting the multi-objective manufacturing tolerance analysis of the absolute optimal configuration under geometric deviations of up to ± 20 nm.

The fabrication tolerance analysis shown in Figure 13 evaluates the structural robustness of the optical sensor against imperfections inherent in photolithography and nanoscale etching processes [39]. By applying artificial geometric deviations of up to ±20 nm relative to the global optimal configuration, the quality factor and sensitivity profiles demonstrate remarkable stability. Although a maximum geometric deviation of ±20 nm induces a slight parabolic decline—caused by a subtle breaking of the cavity’s symmetry—the quality factor remains firmly above 6000, and the sensitivity stays above 5600 nm/RIU. This minimal degradation in optical performance confirms the high tolerance of the GaAs hollow nano-disk resonator to industrial manufacturing variations. The device thus stands out as a highly viable and reliable architecture for large-scale deployment on microfluidic chips for medical diagnostics.

Comparing the data in Table 5, it is clear that the optimized configuration is better than the conventional approaches described in the literature. A review of the different design approaches shows that the traditional physical trade-off between sensitivity and quality factor is consistently encountered by the classical methodologies.

One significant drawback of methods based on discrete sweeps or empirical manual adjustments is that, although they are successful in obtaining a high quality factor through efficient optical confinement, their spectral sensitivity drastically decreases [40]. Conversely, designs relying on numerical analysis or basic deep learning manage to increase sensitivity but suffer from a significant broadening of the resonance peak, which degrades overall resolution and limits the attainment of a competitive figure of merit.

Moreover, the hollow nano-disk structure developed in this work offers the distinct advantage of overcoming this technological bottleneck. By simultaneously combining ultra-high sensitivity with a restored quality factor, the hybrid inverse optimization framework achieves a record-breaking figure of merit. This synergy leads to a drastic reduction in the detection limit, indicating that, for the specific hollow nano-disk resonator investigated here, the hybrid AI-assisted optimization framework achieved a more favorable sensitivity–quality factor trade-off than the discrete-sweep and empirical baselines considered in this study. We note that conventional design and optimization methods remain a valid and often sufficient route for applications where the performance requirements are less demanding, and the choice of design methodology should ultimately be guided by the specific operational and clinical requirements of the target application. Integrating our optical sensor into an automated centrifugal microfluidic platform [41] will enable the automated analysis of HeLa cells. This ’sample-to-answer’ architecture handles the sample from injection through to final optical detection.

## 5. Conclusions

This work presented a hybrid artificial intelligence-driven optimization framework for the inverse design of a two-dimensional photonic crystal optical sensor dedicated to the early detection of cervical cancer (HeLa cells). The proposed architecture combines a hollow-disk resonant cavity with a Random Forest-based parameter screening approach, a multilayer artificial neural network (ANN-MLP) surrogate model, and Particle Swarm Optimization (PSO) to efficiently explore the design space and identify the optimal geometric configuration.

Compared with the conventional FDTD-based parametric design, the proposed optimization strategy substantially enhanced the optical sensor performance. The optimized device achieved a maximum sensitivity of 5676.67 nm/RIU, a quality factor of 6164.5, a figure of merit of 17,739.59 RIU^−1^, and a detection limit of $5.64\times 10^{-5}$ RIU, demonstrating a significant improvement in sensing performance while maintaining excellent spectral selectivity. Furthermore, the manufacturing tolerance analysis confirmed that the optimized structure preserves stable performance under geometric deviations of up to ±20 nm, highlighting its robustness for practical nanofabrication.

Beyond improving a single optical sensor design, this study demonstrates the potential of integrating machine learning with electromagnetic simulations to accelerate the inverse design of nanophotonic devices. The proposed framework reduces the computational burden associated with exhaustive FDTD parameter sweeps while providing an efficient methodology for identifying high-performance photonic structures. Consequently, the presented approach can be extended to the optimization of a wide range of photonic crystal optical sensors and integrated optical sensing platforms. While the proposed ANN-PSO framework demonstrably improved the sensing performance of the specific architecture studied here, we do not claim that AI-based optimization is a strict requirement for achieving clinically adequate optical sensor performance in general; rather, it offers an efficient means of exploring a nonlinear design space that may be impractical to fully characterize through exhaustive parametric sweeps alone.

Although the present study is based on numerical simulations, the obtained results provide a strong foundation for future experimental realization. Future work will focus on device fabrication, experimental validation using real biological samples and microfluidic platforms, robustness analysis under fabrication imperfections and environmental noise, and the extension of the proposed AI-assisted optimization framework to multiplex biosensing and other biomedical diagnostic applications.

## Figures and Tables

**Table 2:** The designed optical sensor 2D-FDTD performance.

	Performance	RI (n)	$\lambda_{r}$ (nm)	$\Delta_{\lambda_{r}}$ (nm)	$\Delta_{n}$	Sensitivity S (nm/RIU)	FWHM (nm)	Quality Factor (Q)	Detection Limit DL (RIU)	FOM (RIU^−1^)
Cells										
Without bio-sample		1	1851.4	-	-	-	0.5	3702.8	-	-
Normal cells 0%		1.368	1859.2	-	-	-	0.5	3718.4	-	-
Low-stage cancer 25%		1.374	1862.8	3.6	0.006	600	0.5	3725.2	$8.33\times 10^{-4}$	1200.48
Moderate-stage cancer 50%		1.380	1866.3	7.1	0.012	591.67	0.5	3732.2	$8.45\times 10^{-4}$	1183.43
Advanced-stage cancer 75%		1.386	1869.8	10.6	0.018	588.89	0.5	3739.2	$8.49\times 10^{-4}$	1177.85
Severe-stage cancer 100%		1.392	1873.1	13.9	0.024	579.17	0.5	3745.4	$8.63\times 10^{-4}$	1158.34

**Table 3:** Initial evaluation of optical sensor performance using the Random Forest method.

Biosample Stage	RI (n)	$\Delta_{n}$	$\lambda_{R}$ (nm)	$\Delta \lambda_{R}$ (nm)	Sensitivity (S) (nm/RIU)	Quality Factor (Q)	FWHM (nm)	FOM (RIU^−1^)	Detection Limit DL (RIU)
0% (Normal)	1.368	–	1842.5	–	–	4848.68	0.38	–	–
25% (Low)	1.374	0.006	1856.12	13.62	2270	4884.52	0.38	5973.68	$1.67\times 10^{-4}$
50% (Moderate)	1.380	0.012	1870.22	27.72	2310	4921.63	0.38	6078.95	$1.64\times 10^{-4}$
75% (High)	1.386	0.018	1884.92	42.42	2356.67	4960.31	0.38	6201.76	$1.61\times 10^{-4}$
100% (Severe)	1.392	0.024	1900.1	57.6	2400	5000.26	0.38	6315.79	$1.58\times 10^{-4}$

**Table 4:** Analysis of optical sensor performance based on the ANN-PSO algorithm.

Biosample Stage	$\lambda_{R}$ (nm)	Δ$\lambda_{R}$ (nm)	Sensitivity (S) (nm/RIU)	Quality Factor (Q)	FWHM (nm)	FOM (RIU^−1^)	Detection Limit DL (RIU)
0% (Normal)	1836.400	–	–	6121.33	0.300	–	–
25% (Low)	1868.520	32.12	5353.33	6146.45	0.304	17,609.64	$5.68\times 10^{-5}$
50% (Moderate)	1901.884	65.48	5456.67	6135.10	0.310	17,602.16	$5.68\times 10^{-5}$
75% (High)	1936.568	100.17	5565.00	6128.38	0.316	17,610.76	$5.67\times 10^{-5}$
100% (Severe)	1972.640	136.24	5676.67	6164.50	0.320	17,739.59	$5.64\times 10^{-5}$

**Table 5:** Comparative performance benchmarking of the proposed 2D-PhC optical sensor against recent fully-quantified literatures.

Author Reference	Analyte Target	Type of Resonance	Sensitivity (S) (nm/RIU)	Quality Factor (Q)	FOM (RIU^−1^)	Detection Limit DL (RIU)	Optimization Method
Rizk, S et al. [19] (2025)	Tumor cells	Eye-shaped cavity	148.5	3120	450.2	$2.15\times 10^{-4}$	Manual adjustment
Roshani, S et al. [20] (2025)	Glycerol–water mixture measurement	Ring waveguide	89.9	5233	299.66	$3.33\times 10^{-3}$	ANN regression
Balaji, et al. [21] (2024)	HeLa multi-cancer	Triangular defect	600	15,000	1200.48	$8.33\times 10^{-4}$	Discrete sweeps
Karami, et al. [22] (2026)	HeLa multi-cancer	Coupled nano-cavity	3121	5412	6340.3	$5.53\times 10^{-5}$	Computational analysis
Nipun, et al. [23] (2026)	Skin cancer	Compact resonator	1245.6	2150	2541.2	$1.41\times 10^{-4}$	AI-driven surrogate optimization
Molaei Yeznabad, A. and Bahador, H. [24] (2025)	Cancer cell detection	—	692.8	5870.11	2561.5	—	Manual adjustment
This work	Cervical cancer (HeLa)	Hollow nano-disk	5676.67	6164.5	17,739.59	$5.64\times 10^{-5}$	Hybrid ANN-PSO

## Data Availability

The original contributions presented in this study are included in the article. Further inquiries can be directed to the corresponding author.
